# A Plantar Pressure Sensing System with Balancing Sensitivity Based on Tailored MWCNTs/PDMS Composites

**DOI:** 10.3390/mi9090466

**Published:** 2018-09-13

**Authors:** Xuefeng Zhang, Running Chai, Haitao Wang, Xiangdong Ye

**Affiliations:** 1 Shaanxi Key Laboratory of Nano Materials and Technology, Xi’an University of Architecture and Technology, Xi’an 710055, Shaanxi, China; chairunning0128@gmail.com (R.C.); yexiangd@xauat.edu.cn (X.Y.); 2 School of Mechanical and Electrical Engineering, Xi’an University of Architecture and Technology, Xi′an 710055, Shaanxi, China; wanghaitao@xauat.edu.cn

**Keywords:** plantar pressure sensor, composites, multi-walled carbon nanotubes (MWCNTs), piezoresistive

## Abstract

This paper presents a flexible plantar pressure sensor with a simple structure and easy accessibility, suitable for everyday use. In this study, the design, fabrication, and characteristics of both the composite and the sensor were involved. By using the solution method, the piezoresistive composite was fabricated by uniform dispersion of multiwall carbon nanotubes (MWCNTs) into the polydimethylsiloxane (PDMS) matrix. The proposed sensor consists of eight sensing elements with a laminated structure. The upper layer is a sensing layer made of the piezoresistive composite, and the lower layer is a flexible printed circuit-board working as electrodes. A particular design of sensing elements was carried out by using different doping concentrations according to arrangement positions under the feet to obtain balancing sensitivity. A signal processing system to convert the variable resistance signal into voltages by the current-to-voltage method was designed. Experimental results prove that the designed sensor shows a repeatable response with a sensitivity of 11.5 mV/kPa within the range of 265 kPa. Also, an actual application verifies that the designed plantar pressure sensor can measure the pressure under the foot and can be used for gait detection and disease diagnosis purposes.

## 1. Introduction

Plantar pressure is an essential biomechanical parameter which can be applied in many situations, from daily life to industrial production. For instance, conventional analysis of plantar pressure in the healthcare domain relates to diabetic foot diagnosis and post-stroke rehabilitation [1,2]. The magnitude and distribution of plantar pressure are considered as factors related to movement and injuries, and thus can be used in training to enhance performance and avoid injury in athletes [3,4,5]. Also, the control of exoskeleton systems for assisting human walking can be realized by the detailed information on the gait acquired from the plantar pressure sensor [6,7]. In a recent study, the pressure under the foot working together with the neural network was found to also be used for avoiding work-related injuries [8]. At the same time, plantar pressure is often helpful in the optimized design of footwear and the insole to get more stable and comfortable products [9]. Among all these applications, the most important purposes are for motion detection and gait analysis [10,11,12,13]. Nonetheless, plantar pressure should be measured correctly and in a timely manner for all of the purposes above.

Nowadays, two different systems can be used to acquire the foot plantar pressure. The first one is the plantar pressure measurement platform system, a great tool for measuring the plantar center of the foot pressure along with the ground reaction force [14]. However, due to its complexity, such a system is only suitable for use in laboratories or hospitals instead of everyday use. The second one is the plantar pressure measurement insole system that can be incorporated into shoes without causing any discomfort or affecting the natural gait by matching its softness, elasticity, and compliance [15,16]. With these systems, plantar pressure can be measured during different tasks performed in different environments and time scales, and both systems demand for sensors with high performance and a compact structure.

The piezoresistive effect and capacitive principles are the most frequently used methods in plantar pressure measurement [15]. An excellent plantar pressure sensor should be flexible, thin, and have relatively high sensitivity in a broad enough pressure range, with good repeatability and long durability. Some published works have demonstrated the potential of capacitive pressure sensors for plantar pressure measurement because of their good sensitivity and linearity [17]. However, capacitive sensors are vulnerable to external electromagnetic interferences and to losing their precision [18], whereas resistive sensors have inherent advantages, such as easy interfacing with peripheral circuits, being less susceptible to noise, and their ability to work well in all configurations [19].

Until now, there have been considerable efforts placed in the design and development of piezoresistive sensors for plantar pressure measurement. For example, researchers used silicon stress sensors realized by microelectromechanical system (MEMS) techniques to improve the accuracy of the measurements [10,11]. To further reduce the size of the insole, especially the thickness, the development of plantar pressure sensors took advantage of the piezoresistive effect of conductive composites [16]. In the past few years, different composite materials have been developed to realize plantar pressure sensors with optimized performance. By incorporating carbon nanotubes into a polydimethylsiloxane (PDMS) matrix, a pressure sensor system for plantar pressure monitoring has been successfully realized [12]. The reduction of graphene oxide has been realized and used to get a pressure sensor with higher sensitivity and a broader measurement range for gait analysis [13,20]. These studies improved the sensitivity or the measurement range of all the pressure sensors in the insole systems.

As far as we know, all of the plantar pressure measuring systems in the reported literature to date have adopted uniform sensing elements with the same measuring range and sensitivity. However, the pressure of different regions under the foot is obviously different. To improve the measuring performance of the plantar pressure sensing system, the sensing elements in an insole should not be identical because of the different distribution of pressure under the foot. In this paper, an insole based on the piezoresistive effect of a multiwall carbon nanotube (MWCNTs)-doped PDMS composite was developed for plantar pressure measurement. Sensing elements in different regions of the insole adopt dissimilar composites with different doping ratios to optimize the sensitivity and measurement range. Moreover, a straightforward method for fabricating sensing elements was designed to improve the repeatability and stability. The entire sensor was interfaced with a resistance readout circuit to obtain the pressure-to-voltage relation with applied pressure for use in practical applications.

Firstly, the paper briefly introduces the main techniques for plantar pressure measurement, including the various sensors used to measure the pressure under the foot. Then, the fabrication of the composites, pressure sensor, and the signal processing system are described. After that, the experimental results and discussion are presented to validate the utility of the presented plantar pressure measurement system. Finally, conclusions are drawn in the last section.

## 2. Material Preparation and Characterization

### 2.1. Material Preparation

**Figure 1:**
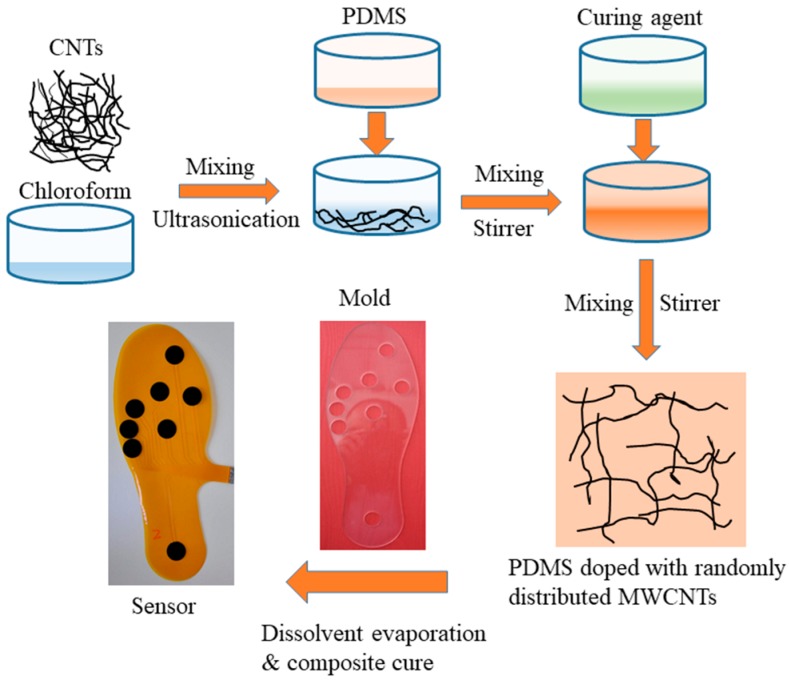
Schematic of the processing procedure for preparing a multiwall carbon nanotubes (MWCNTs)/polydimethylsiloxane (PDMS) conductive elastomer and fabricating the plantar pressure sensor.

PDMS has some outstanding features, including excellent mechanical performance, mechanical flexibility, low weight, workability, chemical stability, and chemical inertness, while MWCNTs has a high elastic modulus and excellent electrical properties [21]. MWCNTs should be adequately dispersed within the matrix to build a conductive network, and a high-quality sensor could be achieved by using the particular electrical properties of the network. In this paper, the solution method was used to prepare the MWCNTs/PDMS piezoresistive nanocomposites. A schematic of the procedure for preparing the composite is illustrated in Figure 1. Firstly, the MWCNTs (outer diameter of 10–30 nm, length within 10–30 μm, and purity >90%, sold by Chengdu Organic Chemicals Co. Ltd., Chengdu, China) and chloroform were submitted to ultrasonication at 30 °C for 2 h to mix them thoroughly and obtain the well-dispersed suspension. Secondly, PDMS prepolymer (Sylgard 184, Dow Corning, MI, USA) was added to the mixture and blended with a mechanical stirrer for 30 min. Next, the curing agent was added to the blend (weight ratio of 1:10). Then, after another 30 min of stirring, the mixture was heated on the hot plate at 70 °C to evaporate the chloroform in the fume hood. After that, the mixture was degassed in a vacuum chamber for 30 min to remove the air bubbles. Finally, the glue-like black blend was poured into the mold to cure at 90 °C for 24 h. Fourteen composites with various MWCNTs weight fractions, from 2 wt% to 11 wt%, were prepared according to the above procedure. For each MWCNTs concentration, four samples were fabricated to investigate the uniformity.

### 2.2. Material Characterization

A Field Emission Scanning Electron Microscope (FESEM, JEOL-6700F, Tokyo, Japan) was used to characterize the distribution of CNTs in the fracture surface of the samples. The samples were broken after being cooled with liquid nitrogen. The fractured surface was sputtered by a thin layer of Au to achieve a clear image.

For analyzing the electrical conductivity property of the composite, a high-accuracy digital multimeter (Keysight 34465A, Santa Rosa, CA, USA) was used to measure the resistance of the composite with different CNTs content. Four specimens were tested for every kind of composite, and the average was used to guarantee the measurement precision. The shape of the specimen was a cylinder 1 mm thick, with a diameter of 12 mm.

**Figure 2:**
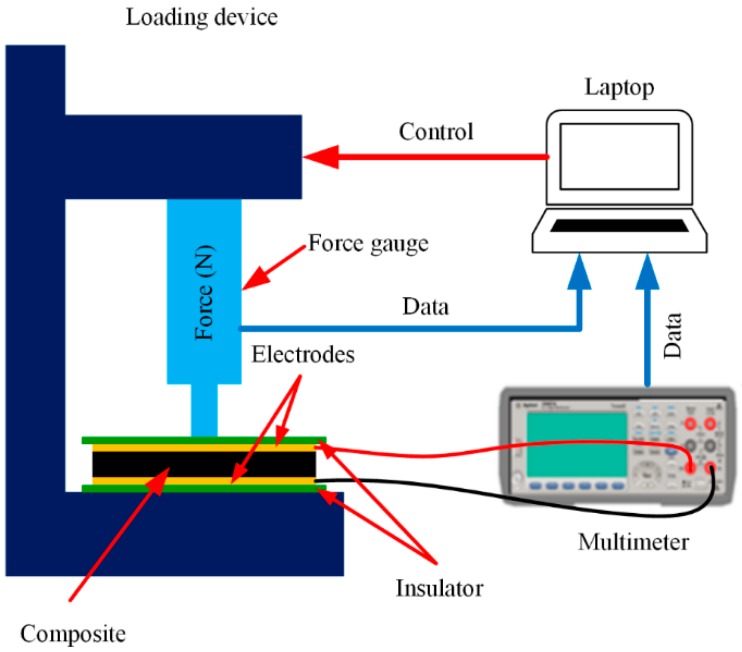
Schematic diagram of the experimental setup.

The piezoresistive property of the composite was tested by the experimental setup shown in Figure 2. The fabricated composite was sandwiched between two Cu electrodes, and two pieces of polyamide insulators suppressed the interface during the measurement. The measurement was carried out by applying a compress force on the specimen by using a vertical motorized test stand (FGS-50E, Nidec-Shimpo Corporation, Kyoto, Japan) for force gauging. A force gauge (FGP-5, Nidec-Shimpo Corporation) was fixed onto the moving stage to measure the loading force (±0.2% of full scale). A digital multimeter measured the electrical resistance of the specimen. When the moving stage moved down and made the force gauge contact with the surface of the specimen, the composite was compressed. As the thickness of the specimen changed, the internal conductive network structure was modified accordingly. This variation of the conductive network structure induced the change in the measured resistance. A host computer controlled the proper loading of the specimen by controlling the stroke of the moving stage and recorded both the force and resistance data.

### 2.3. Morphology of the Composite

**Figure 3:**
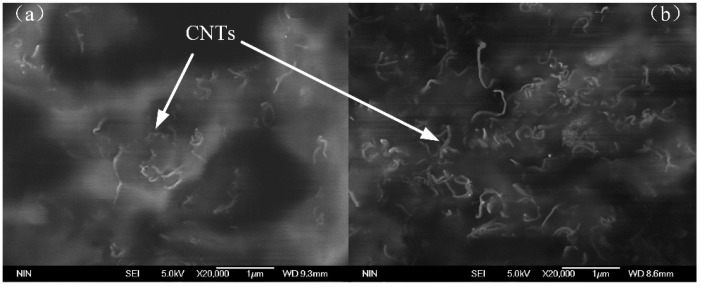
(**a**) Scanning Electron Microscope (SEM) images of the composite with 5 wt% MWCNTs content; (**b**) SEM images of the composite with 6 wt% MWCNTs content.

As the distribution of MWCNTs in the PDMS matrix intrinsically affects the electrical property of the composite, the microstructure of MWCNTs/PDMS composites was investigated first. Figure 3 shows the Scanning Electron Microscope (SEM) images of the section of composites with different MWCNTs content, where MWCNTs are well-dispersed in the PDMS matrix in all the composite specimens. This result can be attributed to the appropriate process of synthesis of the composite (i.e., a powerful ultrasonication) to break down the entangling of the MWCNTs and the choice of an appropriate solvent to dissolve the high-viscosity PDMS polymer matrix.

### 2.4. Electrical and Electromechanical Properties of the Composites

**Figure 4:**
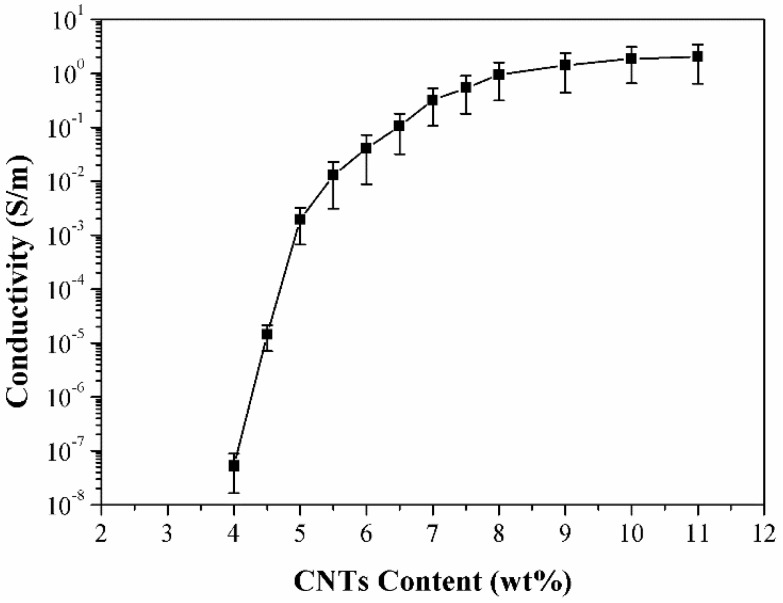
Composite electrical conductivity as a function of MWCNTs content.

The MWCNTs content significantly affected the conductivity of the MWCNTs/PDMS composites [22], and it could be characterized in terms of the conductivity of the composite. Figure 4 shows the curve describing the electrical conductivity as a function of the filler concentration of MWCNTs/PDMS composites. The error bar indicates the variation among the different samples.

As shown in Figure 4, the conductivity of the composite is quite low when the MWCNTs content is lower than 4 wt%. In this situation, the amount of MWCNTs is inadequate to form an effective conductive network in the composite, thus inhibiting the current flux through it. Therefore, the composite is in an insulation state at this stage. When the MWCNTs content is at 4 wt%, a sparse conductive network has been successfully formed in the PDMS matrix—however, the conductivity of the composite is still low. Much denser conductive networks are formed in the composite with an increase in the MWCNTs content, which results in a rise in the conductivity of the composites. When the MWCNTs content has reached 8 wt%, MWCNTs conductive networks have been formed efficiently and the contacts among MWCNTs tend to be saturated and fail to respond to higher conductivity with a further increase in MWCNTs content.

Table 1 shows the comparison of composites’ parameters between previous studies and the study at present. In comparison with the data reported by other researchers, the conductivity of composites in this paper is relatively low [23,24,25]. Such a difference may be attributed to the discrepancy of the MWCNTs and the parameters used in the fabrication process. Both the ratios and the purity of the CNTs used in this paper are lower than that used in other works. On the other hand, the sonication and vigorous stirring in the manufacturing process may have shortened the CNTs and increased the percolation threshold. The results prove that the solution method is feasible for preparing the MWCNTs/PDMS composites, even though there is still room for improving the specific parameters.

**Figure 5:**
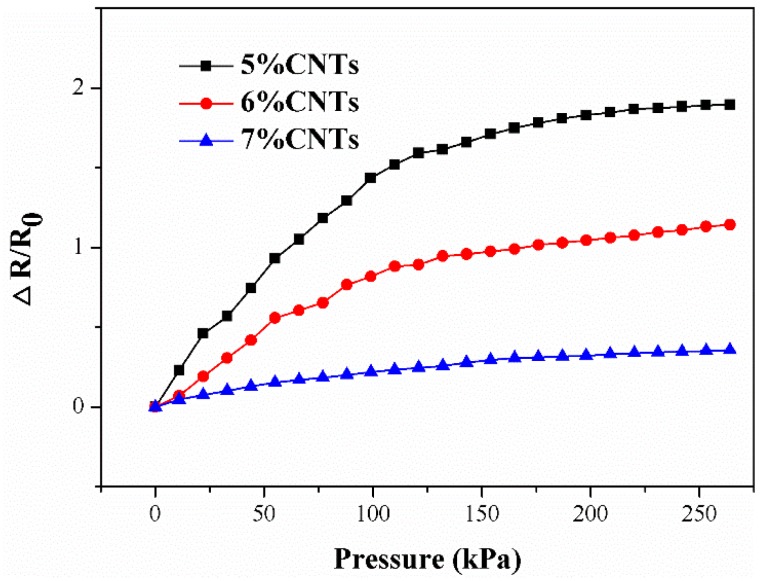
Electromechanical performances composites having different MWCNTs content.

The electromechanical properties of the composite can be characterized in terms of piezoresistivity. The piezoresistive curves of three composites with different MWCNTs contents are presented in Figure 5. *R*_0_ is the initial resistance of the sample before it was compressed; Δ*R* is the difference between the initial resistance and the resistance of the sample after it was loaded. The opposite sides of the samples were pasted with conductive silver paste to eliminate any contact resistance and to achieve a stable output. The electrical resistance of the composites was measured with a variable loading force. The force was applied to the samples in normal directions, causing deformation of the composite that derived from the macromolecular chain of the polymer, which initiated the revolution of the MWCNTs conductive networks because the MWCNTs were embedded in the matrix of polymer and realigned with the polymer chains of the matrix. During the compression, some new connections of the MWCNTs are formed, which introduce additional conductive networks and lead to a decrease in resistance. However, some detachment of the MWCNTs in the conductive networks occurs at the same time, which brings about an increase in resistance. These two mechanisms work simultaneously and result in the collaborative effect of pressure-sensing behavior of the composite. From the piezoresistive curve (Figure 5), the resistance of all the composites increases when they are compressed. It can therefore be concluded that the destructive effect of the MWCNTs conductive networks is dominant in all of these samples. From Figure 5, the composite with the lower MWCNTs content demonstrates higher pressure sensitivity. Specifically, the sample with 5 wt% MWCNTs has a broader variety of electrical resistance than the other two samples under the same loading. From this significant difference, different composites with tailored sensitivity can be used to satisfy diverse plantar pressure measurement ranges at different regions under the foot using various MWCNTs content.

## 3. Sensor Development

### 3.1. Sensor Architecture

**Figure 6:**
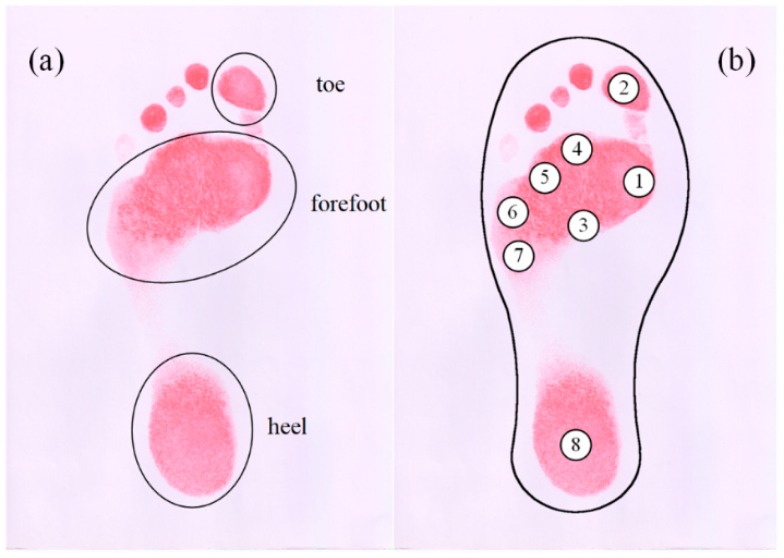
(**a**) Footprint of a bare foot; (**b**) the targeted locations for arranging sensing elements.

For the correct reading of the plantar pressure, the number, arranging locations, and size of the sensing elements should be determined carefully [15]. A colored footprint was used to correctly determine the required number and appropriate location of the sensing elements on an insole. The footprint was captured by pressing a bare foot into red ink and stamping it onto a sheet of white paper (see Figure 6a). From this footprint, three regions could be distinguished: the heel, forefoot, and toes, respectively [26]. After analyzing the footprint, eight pressure concentration regions under the foot were approximately marked by circles and numbers (see Figure 6b). These regions were selected as the locations for the sensing elements arrangement. More specifically, one element was arranged beneath the toe, and another one beneath the heel—the remaining six elements were all arranged under the forefoot.

**Figure 7:**
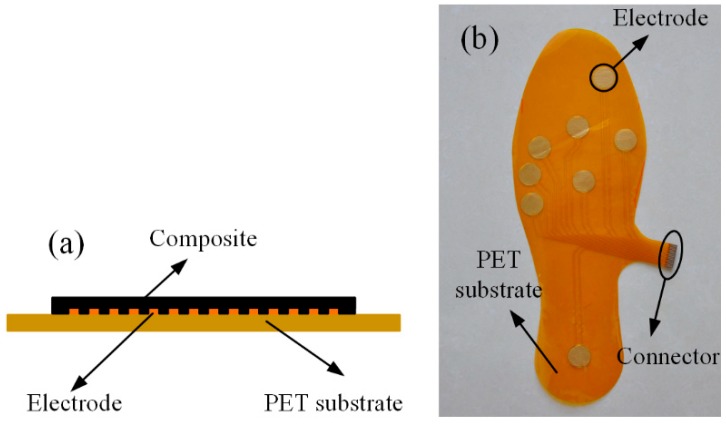
(**a**) Structure diagram of a sensing element (not to scale); (**b**) fabricated flexible insole with the electrodes and connector.

The designed plantar pressure sensor adopts the laminated structure shown in Figure 7a. The upper layer (sensing layer) is made of the composite, and the lower layer (the electrodes) is made using the flexible printed circuit board technique. Eight interdigital electrodes, with a radius of 7.5 mm, were designed and fabricated on a 0.2 mm-thick Polyethylene terephthalate (PET) substrate using the flexible printed circuit process. The electrical connections between sensing elements and peripheral circuit were formed by copper traces created with the electrodes by etching. The fabricated insole, with eight electrodes and conductive traces, is shown in Figure 7b.

### 3.2. Sensor Fabrication

Processing a conductive composite with piezoresistance and, based on that, realizing a flexible pressure sensor prototype for plantar pressure measurement were the ultimate objectives of this study. The conductive composite described in the material preparation section were used to realize the sensing elements on the electrodes of the insole.

The entire processing procedure is shown in Figure 1. By fully incorporating the MWCNTs into the PDMS matrix, the conductive composite was obtained. The MWCNTs/PDMS blend described in the material preparation section was distributed onto the patterned electrodes by a mold that was designed according to the target allocations of electrodes and made of Polymethylmethacrylate (PMMA). After sticking the mold onto the flexible electrode, the blend was poured into the hollows, and unnecessary bits were scratched off by the blade coating method. This action ensured the hollows were filled with the composite and that the upper surface was flat. Finally, the insole was degassed and cured at 80 °C for 2 h. After detaching the mold, the flexible sensor for plantar pressure measurement was achieved. Note that composites with different MWCNTs were used to realize different sensing elements at the various positions for obtaining homogeneous outputs. Specifically, sensing elements numbered 1, 2, and 8, which were able to withstand more considerable amounts of pressure, were realized by the 6 wt% MWCNTs composite, while the other elements were realized by the composite with 5 wt% CNTs. The compact structure and straightforward fabrication of the sensor enabled the fabricated insole pressure sensor to be only 1 mm in thickness. Furthermore, the single-step method to fabricating sensing elements by depositing the composites on the electrodes was very helpful for dealing with the effect of contact resistance.

### 3.3. Signal Processing System

**Figure 8:**
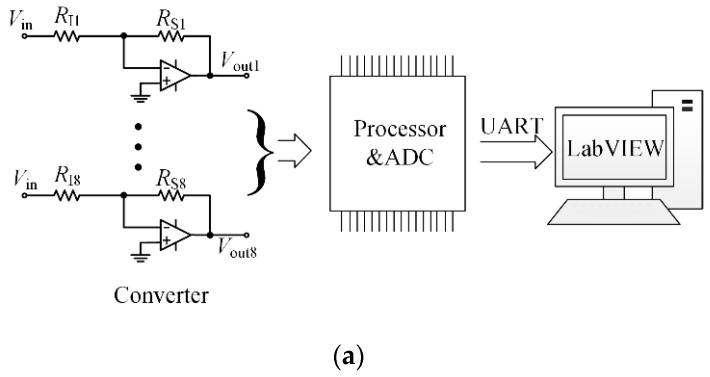

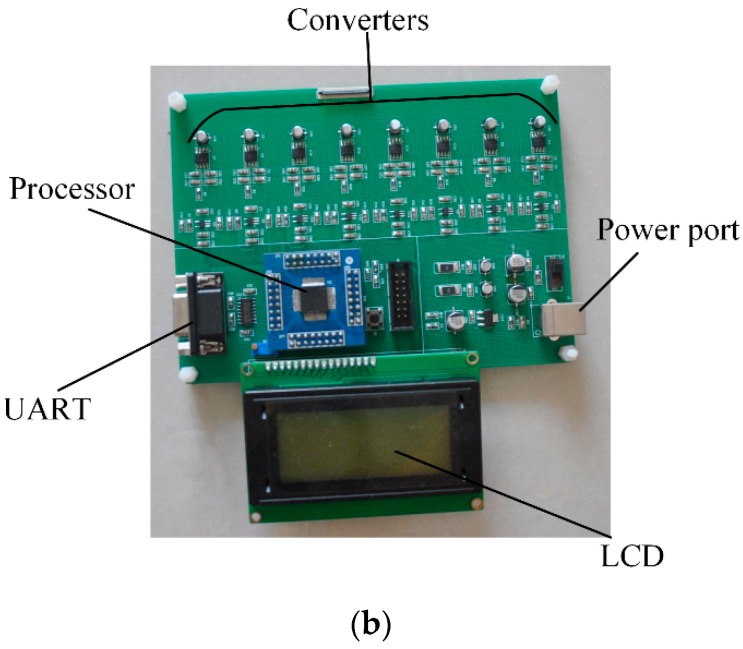
(**a**) Block diagram of the signal processing system; (**b**) the implemented signal processing system.

For convenient use of the sensor for plantar pressure measurement, a signal processing system is desired. Figure 8a shows the schematic diagram of the signal processing system. The system is composed of eight simple resistance-to-voltage converters and a processor. This processor incorporates an analog-to-digital converter and a communication module for communicating with the host computer. The current-to-voltage approach was used to convert the pressure-related variable resistance to a varying voltage signal (see Figure 8a). In this configuration, the resistor *R_Ii_* is the input of the converter; *R_SIi_*, that is, the resistance of the *i*th sensing element, is connected between the inverting input and the output. Then, the output voltage *V_outi_* of this converter can be described by Equation (1) where *V_in_* is the driving voltage. Since the elements 1, 2, and 8 had higher MWCNTs content, they showed a weaker response with equal pressure, compared to the other five elements. Therefore, different input resistances were adopted to get higher levels of measurement accuracy with the desired sensitivity.

(1) $$V_{outi}=-\frac{R_{SIi}}{R_{Ii}}\times V_{in}\;$$

According to Equation (1), to obtain a positive output voltage, a negative driving voltage *V_in_*, is essential. Therefore, dual-sided power supplies for the amplifier are necessary. As a result, the output voltage *V_outi_* is proportional to the resistance of the sensing element, meaning the output voltage changes while the sensing element is loaded. Considering its accuracy, stability, and power consumption, the high-precision operational amplifier OPA4277PA (Texas Instruments, Dallas, TX, USA) was selected to realize the converters. Moreover, the ultra-low-power and high-performance microcontroller MSP430F149 (Texas Instruments) was used to implement the analog-to-digital conversion and data transmission. The implemented signal processing system is shown in Figure 8b. A customized GUI (Graphical User Interface) written in LabVIEW (2014, National Instruments Corporation, Austin, TX, USA) was used to present the plantar pressure data intuitively on the host computer.

## 4. Results and Discussion

### 4.1. Characterization of Sensing Elements

The experimental setup shown in Figure 2 was also used to test and characterize the performance of the plantar sensor. In addition to the setup, the signal processing system described above was adopted together. The signal processing system captured and converted the changes of electrical resistance of the sensor into a voltage signal and uploaded it to the host computer through a serial port for storage and further processing. The experimental setup produced a series of stepwise pressures ranging from 0 to 265 kPa to actuate the sensing elements.

**Figure 9:**
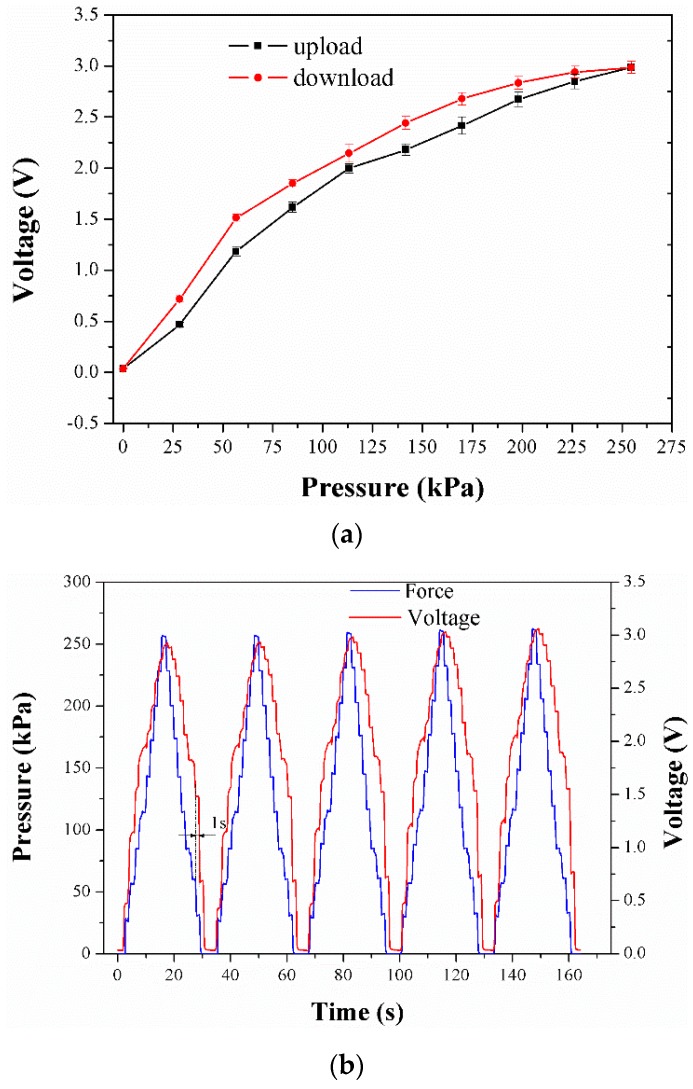
(**a**) Characteristic curve of sensing element 2; (**b**) output of sensing element 2 in five-cyclic loading.

The output curve of sensing element 2 with the variable load is shown in Figure 9a. The result describes the output voltage of the sensing element as a function of the actuating pressure. Specifically, the output of the sensing element increases with the rising pressure because of the piezoresistivity of the composite and the configuration of the signal processing system. In the figure, the least-square fitting method was used to obtain a linear model of the calibration data of the five cycles of loading and unloading, and the error bars were estimated. All the eight sensing elements have been calibrated by the same procedure as shown in Figure S1. Moreover, the characteristics in terms of linearity, hysteresis, and repeatability of all of the sensing elements were calculated based on the least-square fitting results and are listed in Table 2. The table highlights that all the sensing elements produce an output with high repeatability, and the sensitivity is up to 11.5 mV/kPa. However, comparatively weak linearity and visible hysteresis can also be observed. These two issues may have caused the low accuracy in the plantar pressure measurement, which might have been partly due to the calibration apparatus adopted in the experimental setup which could not maintain a constant force during the calibration. On the other hand, the viscoelastic property of the PDMS matrix will be responsible for the problem too. In addition, it is noted that a slight difference in performance of all the sensing elements exists, which can be attributed to the non-identical distribution of the MWCNTs in the different sensing elements.

Figure 9b shows the output curve of sensing element 2 in the five-cyclic-load procedure. The output of the sensing element follows close behind the varying pressure in the whole measurement range. For all the applied loads, the sensing element shows similar piezoresistive behavior from the first cycle to the last. All these indicate that stable and recoverable conductive networks have been successfully constructed during the cyclic compression process. Both the high repeatability of the sensing element and the uniformity among all eight sensing elements are the result of the MWCNTs’ uniform dispersion and single-step method to fabricating sensing elements.

However, a time delay can be found between the actuating load and the output. The maximum delay of about 1 s is located in the download process, and inhibits high-accuracy measurement of the plantar pressure. It is inferred that both the hysteresis and time delay originate from the natural viscoelasticity of the PDMS matrix. Therefore, the designed plantar pressure sensor is not suitable for applications which are strict in real time. The novel material design and innovative structural design of the sensing element should be considered for addressing this problem efficiently [27,28,29].

### 4.2. Application of the Plantar Pressure Sensor

**Figure 10:**
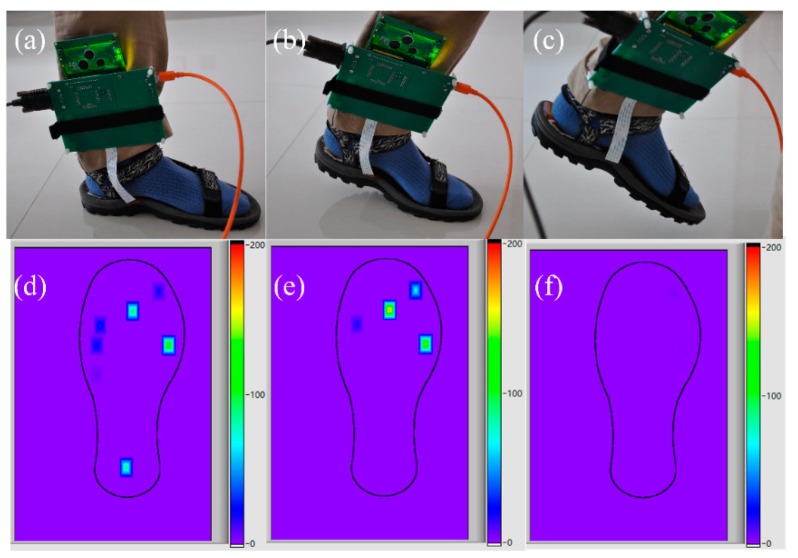
(**a**) Picture of the foot flat; (**b**) picture of the heel off; (**c**) picture of the midswing; (**d**) plantar pressure of the foot flat; (**e**) plantar pressure of the heel off; (**f**) plantar pressure of the midswing.

An experiment was carried out to verify the efficiency of the designed sensor for measuring the plantar pressure during walking. The footstep’s main phases, that is, foot-flat, heel-off, and midswing, are shown in Figure 10.

**Figure 11:**
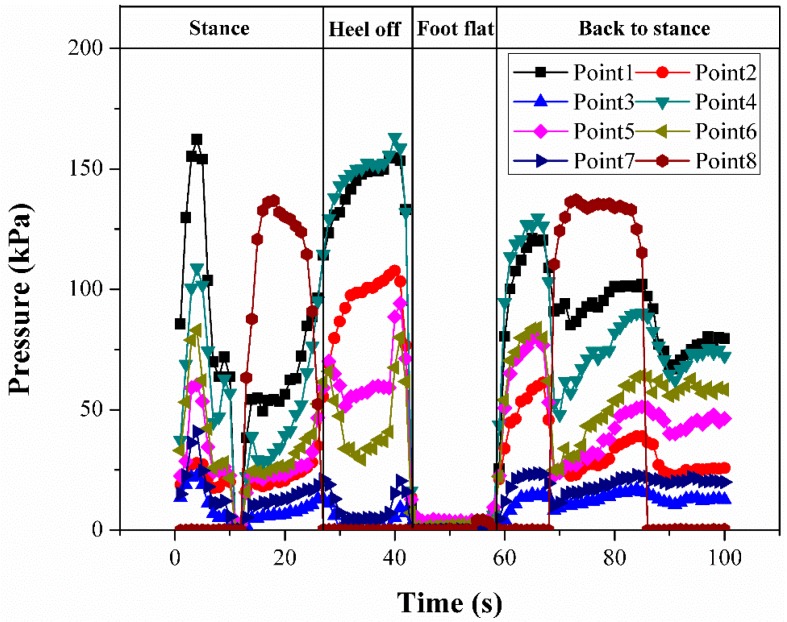
Plantar pressures of all sensing elements during a step.

When a person stands still, the body weight is evenly distributed over the whole foot, as in Figure 10a. During the dynamic gait phase, the body weight is correspondingly supported by different parts of the foot (see Figure 10b,c), and the distribution of plantar pressure reflects this transformation intuitively. Figure 10d–f show the corresponding plantar pressure distribution of these three phases in a standard step, while the specific pressure values of all the sensing elements during a standard step phase are shown in Figure 11. The resulting pressures can be further processed for extracting valuable information for practical purposes.

The experimental results proved that cooperation of the designed sensor with the signal processing system can sense plantar pressure. The developed pressure measurement system offers a more effective solution for plantar pressure measurement, especially for a slack pace.

## 5. Conclusions

In this paper, a piezoresistive sensor based on the MWCNTs/PDMS composite was developed for plantar pressure measurements. The sensing material preparation, structure design, fabrication process of the sensor, and the signal processing systems were presented. In addition, the sensing material was characterized and the sensing mechanism was illustrated. The designed sensor was characterized in terms of linearity, hysteresis, and repeatability. Finally, the proposed plantar pressure sensor has been successfully used to implement a standard step plantar pressure measurement. The results indicate that the proposed device can be successfully applied in plantar pressure measurements, and it has potential applications in gait detection and disease diagnosis. Future studies will focus on improving the dispersion quality of the MWCNTs in the PDMS matrix and simplifying the signal processing system for more convenient, practical applications.

## Figures and Tables

**Table 1:** Parameters of the carbon nanotubes’ (CNTs) PDMS composites.

	Purity	Diameter	Length	Ratio	Percolation Threshold
Ref [23]	90%	5–15 nm	10–20 μm	667–4000	0.13 vol%
Ref [24]	95%	15 nm	40–110 μm	2667–7333	0.045 wt%
Ref [25]	98%	10 nm	3–6 μm	300–600	0.625 ± 0.100% *w*/*w*
This work	90%	10–30 nm	10–30 μm	333–3000	<4 wt%

**Table 2:** Performance parameters of the sensing elements.

	E1	E2	E3	E4	E5	E6	E7	E8	Ref. [30]
Linearity (%)	21.85	16.22	13.09	23.29	19.12	12.99	23.18	23.62	≤±10
Hysteresis (%)	12.75	11.50	13.81	8.00	18.11	8.43	8.57	22.06	≤15
Repeatability (%)	3.91	2.60	0.86	1.59	1.29	1.48	1.57	1.00	≤5

## Supplementary Materials

The following are available online at http://www.mdpi.com/2072-666X/9/9/466/s1, Figure S1: Calibration curves for all sensing elements.
