# Supplementary material for: A Plantar Pressure Sensing System with Balancing Sensitivity Based on Tailored MWCNTs/PDMS Composites
Source: Micromachines (Basel). 2018 Sep 13;9(9):466. doi: 10.3390/mi9090466 (PMC6187450; doi:10.3390/mi9090466)
Supplement: Supplementary file 1 [file micromachines-09-00466-s001.pdf]

micromachines

Supplementary Materials: A Plantar Pressure Sensing System with Balancing Sensitivity Based on Tailored MWCNTs/PDMS Composites

Xuefeng Zhang *, Running Chai, Haitao Wang and Xiangdong Ye

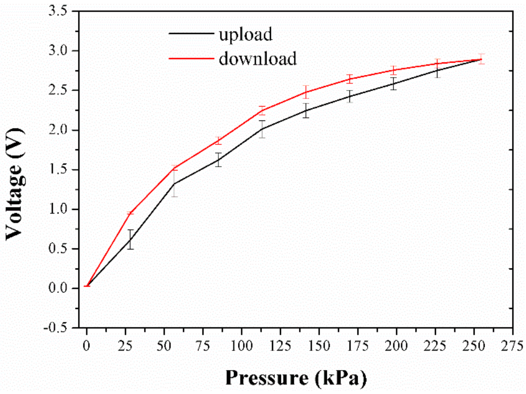

Element 1

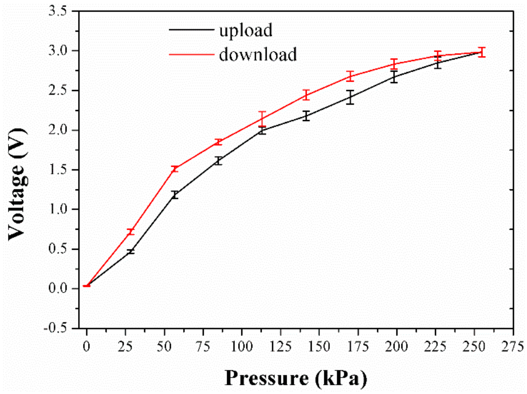

Element 2

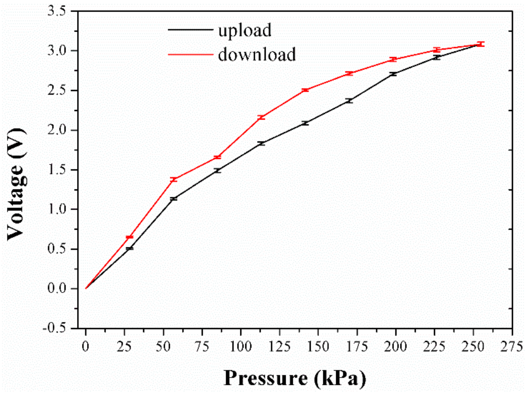

Element 3

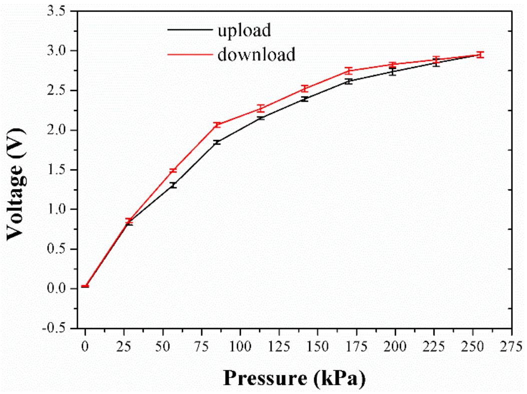

Element 4

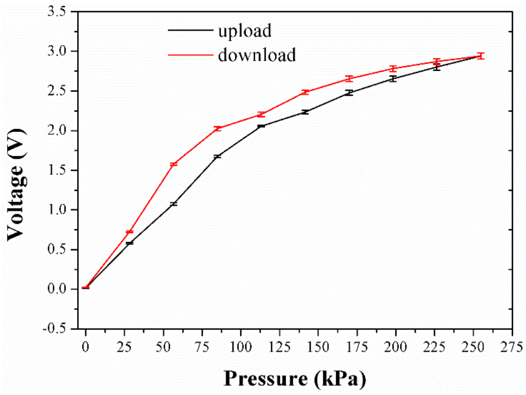

Element 5

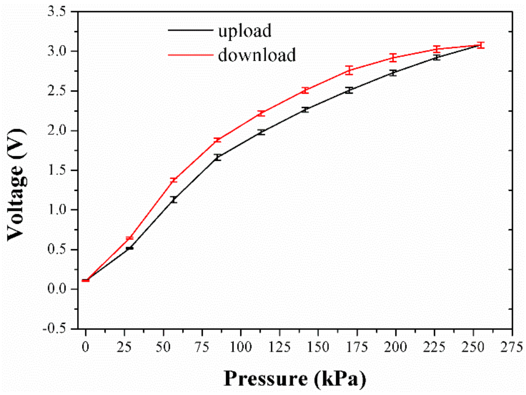

Element 6

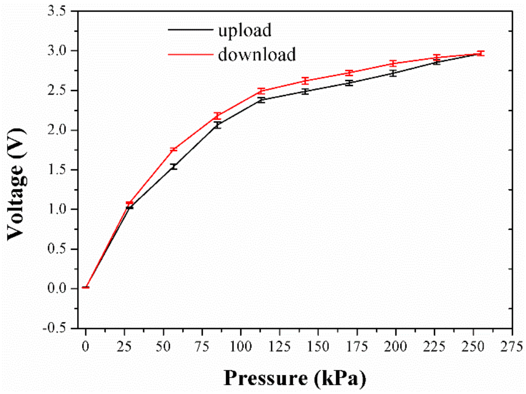

Element 7

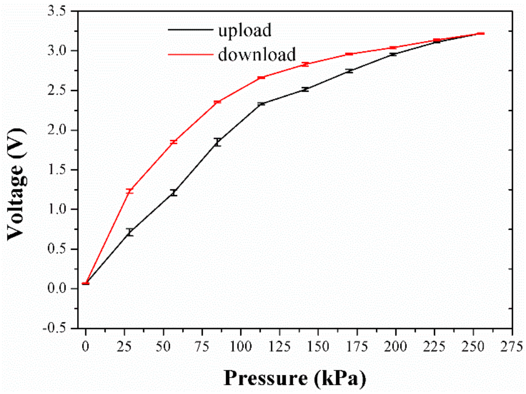

Element 8

Figure S1. Calibration curves for all the sensing elements.
